# Adaptive Fabrication of Electrochemical Chips with a Paste-Dispensing 3D Printer

**DOI:** 10.3390/s24092844

**Published:** 2024-04-29

**Authors:** Ten It Wong, Candy Ng, Shengxuan Lin, Zhong Chen, Xiaodong Zhou

**Affiliations:** 1 Institute of Materials Research and Engineering, A*STAR (Agency for Science, Technology and Research), 2 Fusionopolis Way, #08-03, Innovis, Singapore 138634, Singapore; wongti@imre.a-star.edu.sg; 2 School of Materials Science & Engineering, Nanyang Technological University, Block N4.1, Nanyang Avenue, Singapore 639798, Singapore; candyng107@gmail.com (C.N.); aszchen@ntu.edu.sg (Z.C.); 3 Residues and Resource Reclamation Centre (R3C), Nanyang Environment and Water Research Institute, Nanyang Technological University, 1 Cleantech Loop, Clean Tech One, Singapore 637141, Singapore; lin.shengxuan@gmail.com

**Keywords:** electrochemical (EC), 3D printing, disposable EC chip, sensing, additive manufacture

## Abstract

Electrochemical (EC) detection is a powerful tool supporting simple, low-cost, and rapid analysis. Although screen printing is commonly used to mass fabricate disposable EC chips, its mask is relatively expensive. In this research, we demonstrated a method for fabricating three-electrode EC chips using 3D printing of relatively high-viscosity paste. The electrodes consisted of two layers, with carbon paste printed over silver/silver chloride paste, and the printed EC chips were baked at 70 °C for 1 h. Engineering challenges such as bulging of the tubing, clogging of the nozzle, dripping, and local accumulation of paste were solved by material selection for the tube and nozzle, and process optimization in 3D printing. The EC chips demonstrated good reversibility in redox reactions through cyclic voltammetry tests, and reliably detected heavy metal ions Pb(II) and Cd(II) in solutions using differential pulse anodic stripping voltammetry measurements. The results indicate that by optimizing the 3D printing of paste, EC chips can be obtained by maskless and flexible 3D printing techniques in lieu of screen printing.

## 1. Introduction

Electrochemical (EC) detection is a powerful tool that satisfies the increasing demand for simple, low-cost, and rapid analysis of raw samples with high specificity [1,2,3,4,5]. Various electroanalytical detection methods, including potentiometric, voltammetric, amperometric, and electrochemical impedance spectroscopy, have been developed for different applications, and one of the examples is the voltammetric detection of heavy metal ions [5,6,7,8,9,10,11,12]. For applications such as heavy metal detection, on-site operation with a portable device is highly desired, and disposable EC chips are used in these devices, which are fabricated by the 3D printing of electrodes [13,14,15,16,17]. The advantage of using 3D printing to fabricate EC chips is that—after 3D printing—the fluidic channel with liquid control can be directly printed on the electrodes with no bonding process to form a lab-on-a-chip device.

The 3D printing of electrodes can be categorized as solid material printing and paste material printing [18,19,20,21,22,23]. Electrodes can be printed using conductive filament materials such as carbon black/acrylonitrile butadiene styrene, carbon nanofiber/graphite/polystyrene, graphene/polylactic acid (PLA), and carbon black/PLA. These electrodes are easily integrated into an EC cell for biosensing [13,14,15,16,17,22,23]. However, these electrodes have relatively large sizes and thicknesses, and usually, only one type of conductive material is printed. Metal electrodes are also printed by selective laser melting for stainless steel, which can be further surface-modified with gold or bismuth. The 3D-printed metal electrode functions as a working electrode, a platinum electrode is used as an auxiliary electrode, and an Ag/AgCl electrode is used as the reference electrode [24,25,26]. In this case, 3D-printed metal electrodes cannot form a disposable EC chip. Another method for forming electrodes involves coating and drying fabric materials, weaving them into electrode patterns on flexible materials. However, they have not been used for EC detection as yet [27,28,29].

Screen-printed EC chips offer the advantage of being able to print 2–3 layers of pastes, allowing the working electrode, auxiliary electrode, and reference electrode to be printed on an inexpensive, finger-sized, disposable EC chip. This replaces traditional bulky electrodes. These chips are lightweight, user-friendly, and disposable, consuming a small amount of solvent sample for detection, thus enhancing the portability of EC detections [30]. Screen printing involves squeezing the paste through a patterned mask made of a metal sheet to produce EC chips in high volumes at low cost and with high reproducibility [31]. Carbon-based [32,33], metal-based (gold and silver) [34,35], and film-coated (such as bismuth and gold film) [36,37] EC chips have been reported for different applications. However, a specific and relatively expensive mask is made for each layer of the pattern, which limits the flexibility of screen printing. Hence, in this work, we seek a flexible and low-cost 3D printing method to fabricate EC chips.

Direct paste printing methods for electronic circuits, such as roll-to-roll printing [38,39], inkjet printing [40,41,42], electrohydrodynamic jet printing [43,44,45], and aerosol jet printing [46] are used for printing ultrafine electrodes at a few micrometers for low viscosity (within 2200–4200 cP) paste [45,47]. In terms of higher viscosity pastes for electrodes, dispensing printing is the most suitable technique. In our work, we demonstrate a method to use a conventional dispensing 3D printer to print electrodes. The three-electrode EC chip was designed and 3D-printed on a plastic substrate with a silver paste (2000–5500 cP) and let dry in the air. Subsequently, the substrate was printed with a layer of carbon paste (3100–5800 cP) on a silver layer and baked for 1 h in a 70 °C oven. During printing, challenging issues such as tube bulging (or even bursting), nozzle clogging, paste dripping, and paste accumulation were resolved by selecting suitable tubing and nozzle materials. Additionally, adjustments to the printing process, such as retracting the paste prior to dripping and regularly cleaning the nozzle, further mitigated these problems.

The 3D-printed electrodes were tested with potassium ferrocyanide (K_3_Fe(CN)_6_) in a potassium chloride (KCl) solution for cyclic voltammetry (CV) and heavy metal ions Pb(II) and Cd(II) in acetate buffer solutions using differential pulse anodic stripping voltammetry (DPASV). The printed electrodes demonstrated good reversibility in redox reactions during the CV tests and allowed for the simultaneous detection of Pb(II) and Cd(II) at 200 ppb for both ions. These EC test results were comparable to commercially available screen-printed EC chips. Compared with screen printing, 3D printing is maskless, providing greater flexibility for quick prototyping of customized EC chip designs; it is more cost-effective for small- to medium-volume fabrications; and most importantly, it can print on substrates of any size, which allows for integration with 3D-printed fluidic channels.

## 2. Materials and Methods

### 2.1. Materials

**Figure 1:**
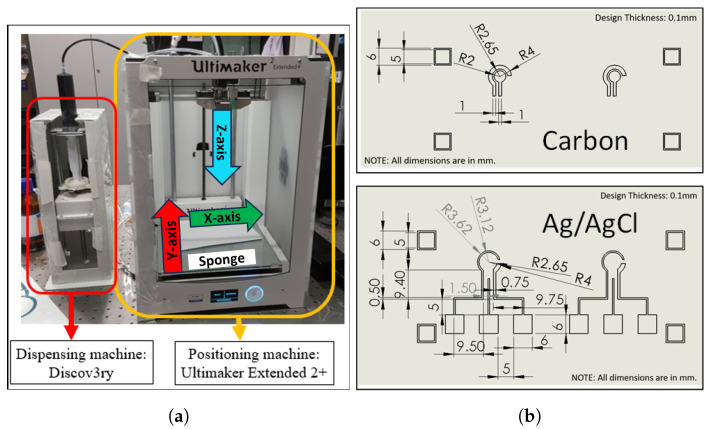
(**a**) Photo of the positioning and dispensing machines for 3D printing, and (**b**) the top (carbon) and bottom (Ag/AgCl) layers of the patterns for EC chips.

The 3D printing machine used to fabricate the EC chips was purchased from Structur3D Printing, Canada. It features an Ultimaker Extended 2+ that acts as the positioning and printing machine, and a Discov3ry dispensing system (Figure 1a). The machine was coupled with a polyethylene tube, a polytetrafluoroethylene (PTFE) tube, and a PTFE-coated needle (as a printing nozzle) with a 0.25 mm diameter supplied by Nordson. The carbon graphite paste (C2030519P4) had a solid content of 39–43%, with 3100–5800 cP viscosity. The normalized sheet resistance was <30 Ω/square at a 16 μm dry film thickness. A silver/silver chloride (Ag/AgCl) paste (C2130809D5) was used, containing 60% silver/40% silver chloride, and 2000–5500 cP viscosity. The normalized sheet resistance was <3 Ω/square at a 25 μm dry film thickness, which was ideal for forming the electrodes. Gray dielectric paste (D2070423P5, formed with polyester, polyvinyl chloride, polycarbonate, or ceramic) was used for insulation. These thress pastes were purchased from Gwent Electronic Materials (acquired by Sun Chemical in 2016) [48], and were used as purchased without dilution. Double-sided adhesive tape (ARcare^®^ 92712), consisting of a 0.5 mil clear polyester film, with both sides covered by 0.7 mil poly(methyl methacrylate) (PMMA) and a layer of 2 mil clear polyester release liner [49], was purchased from Adhesive Research. The substrate for printing the electrodes was a 1 mm thick, 4-inch square PMMA sheet, sourced from Professional Plastics Singapore.

### 2.2. Preparations for 3D Printing

The printing build plate was calibrated each time before printing to keep the distance between the nozzle tip and the substrate at around 0.1 mm.

The key concern when filling the paste into the syringe is to avoid air bubbles, which can cause a discontinuous flow of paste during printing due to uneven force distribution. Thus, the paste was carefully filled using a Zip-lock bag. Firstly, a small hole approximately 2 mm in diameter was cut at the bottom corner of the bag. The paste was slowly pulled into the bag and then squeezed out through the hole into the syringe barrel (see Figure S1, Supplementary Materials). During the filling process, constant shaking or tapping of the bag could help remove any air bubbles in the paste. Once filled, the syringe was installed in the 3D printing machine.

The electrodes were designed using SolidWorks 2016 CAD, as shown in Figure 1b. The electrical conductivity of the Ag/AgCl paste was about 6.4 times higher than that of the carbon paste (when considering the ohm/square values at different dry film thicknesses). Thus, the Ag/AgCl layer was used to form the wire connections and detection pads of the EC chip, although the Ag/AgCl paste was more expensive. As shown in Figure 1b, it is not necessary to have an Ag/AgCl layer below the carbon layer for the auxiliary electrode. However, we designed the auxiliary electrode with two layers to reduce its ohmic resistance, because these two layers can act in parallel, allowing the current to pass through (i.e., the current can move to the detection pad either from the carbon layer or from the Ag/AgCl layer). After 3D printing, the Ag/AgCl layer was covered by the carbon layer and might not have been involved in the EC reaction. In our experiments, we did not see an obvious adverse effect on the EC detection performance of the chip.

The design was saved in the “.stl” format and converted into G-code by running Cura 15.04.5 from Ultimaker (Utrecht, The Netherlands). However, the patterns sliced by Cura were not fully compatible with paste printing, so additional steps were taken by modifying the G-code to optimize the printing results. The modification details are presented in Section 3.1.

### 2.3. EC Chip Fabrication

**Figure 2:**
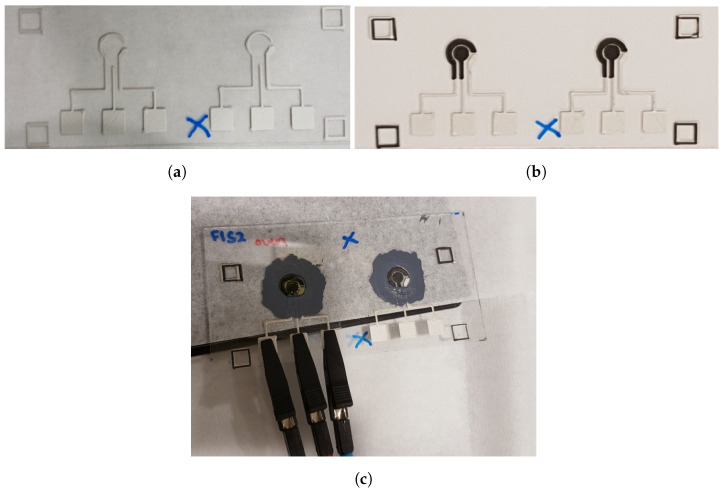
Photos of the 3D-printed (**a**) Ag/AgCl layer (in white, as the first layer), (**b**) the carbon layer (in black, as the second layer), and (**c**) the EC chip insulated by dielectric paste under the EC test.

Our EC chips consist of two layers of electrodes. Firstly, the Ag/AgCl paste was installed into the machine and the modified G-code for the first layer was used for 3D printing. After that, the samples were air-dried. Next, carbon paste was loaded into the machine, and the modified G-code for the 3D printing of the second layer was installed. The carbon was printed on top of the Ag/AgCl layer. Lastly, the samples were heated in an oven at 70 °C for 1 h. The photos of the printed electrodes are shown in Figure 2a,b. For both layers of 3D printing, the nozzle and the printing build plate were kept at an ambient temperature of 25 °C. The nozzle moved in horizontal directions at 6000 mm/min without printing and 300 mm/min during printing; it moved up at a speed of 6000 mm/min and lowered at a speed of 10,000 mm/min (except when the needle tip contacted a sponge for cleaning, at which time it moved at 6000 mm/min).

We printed one layer each of the Ag/AgCl and carbon paste. Moreover, a speed of 300 mm/min was chosen because faster speeds could cause the tube to bulge, while slower speeds made the printing process too slow. The speed of the printing head can be adjusted, provided that the nozzle size is carefully selected to prevent any bulging during the printing process. Printing at different speeds might affect the thickness of the electrodes.

The conducting path of the electrodes should be insulated and leave only the central part for sample loading. Two types of insulation materials were tested: double-sided adhesive tape from Adhesive Research, and dielectric paste from Gwent. A circle matching the size of the central part of the EC chip was drawn using SolidWorks 2016, saved as a “DXF” file, and sent to a Graphtec CE6000-UM-8M2 cutter to cut the shape out of double-sided adhesive tape. The double-sided tape was then adhered to the EC chip, leaving the central part exposed for sample loading. A photo of the fabricated EC chips, insulated by adhesive tape, is shown in Supplementary Materials Figure S2a. The dielectric paste was applied to the conducting path of the electrodes using cotton buds, leaving a central circle open for EC detection. Insulated EC chips with gray dielectric paste are shown in Figure S2b. CV measurements were performed on these fabricated EC chips to determine the best material as the insulating layer.

### 2.4. Performance Tests

EC tests were conducted using the µstat 8000P potentiostat from Metrohm DropSens (Oviedo, Spain). The three electrodes of the potentiostat were connected to the fabricated EC chip using crocodile clips, as shown in Figure 2c. Two types of EC tests were conducted, (1) CV measurements of potassium ferrocyanide; and (2) DPASV measurements of different Pb(II) and Cd(II) concentration solutions.

For the CV test, 100 μL of 5 mM of K_3_Fe(CN)_6_ in 0.1 M KCl solution was pipetted onto the fabricated EC chip. A voltage range from −0.5 V to +0.65 V was scanned at a rate of 0.05 V/s and a step potential of 0.001 V. Three chips were tested, and each chip underwent five cycles of CV tests.

The fabricated EC chips used for the CV tests were washed with fresh deionized water, air-dried, and used in DPASV tests for Pb(II) and Cd(II) in a standard acetate buffer solution. Moreover, 80 μL of a sample solution containing 50 ppb of Pb(II) and 50 ppb of Cd(II) in 0.1 M acetate buffer (pH = 4.5) was pipetted on the top of the electrodes. A deposition potential of −1.4 V was applied to the working electrode for 120 seconds to pre-concentrate the heavy metal ions, followed by recording the voltammogram from −1.3 V to −0.5 V with a step potential of 8 mV, a pulse potential of 0.01 V, a pulse time of 80 ms, and a scan rate of 0.02 V/s. The same test was repeated three times by applying the same experiments to two additional chips. The tests were repeated with concentrations of 200 ppb, 500 ppb, and 1000 ppb of Pb(II) and Cd(II) in 0.1 M acetate buffer, respectively.

## 3. Results and Discussion

### 3.1. Fabrication Process Optimization

As the 3D printing machine was not optimized for high-viscosity paste printing, many issues were encountered and accordingly solved for EC chip printing, as summarized in Table S1.

First, the printing build plate had to be carefully aligned, and the gap between the nozzle and the PMMA substrate was set to 0.1 mm. The nozzle tip could scratch the substrate if the distance was too small, and the printed lines could be disconnected if it was too far (shown in Table S1).

Clogging of needles was a common issue faced in dispensing systems [50]. The needle clogged when the syringe pump pushed the paste into the tube faster than it could be released from the nozzle, building up high pressure in the dispensing system. As a result, the weakest part of the tube bulged and eventually burst if printing continued (shown in Table S1). To overcome this issue, the initially selected polyethylene tube was replaced with a PTFE tube, which offers higher strength. PTFE also has higher surface tension than polyethylene, thereby generating less friction with the paste and reducing tension in the tube. Therefore, the flow of the paste became smoother and less likely to accumulate in the needle. Even if some paste did accumulate by chance, the PTFE tube was tough enough to withstand the pressure required to push the accumulated paste out of the needle and prevent clogging. Paste accumulation inside a needle can occur if the needle is reused and not properly sealed at the tip to prevent the evaporation of solvent into the environment. The humidity of the working and storage environments also affects the smoothness of paste dispensing.

Slicing software Cura was used to generate the G-code from a drawing file in “.stl” format. However, the algorithms of Cura slicing software were designed for printing thermoplastics, making the printing mechanism generated using the Cura software partially incompatible with paste printing. After finishing printing at one position, the printing nozzle moved directly to a new printing position. This movement created unwanted whiskers, which are lines that formed between one printing position and the next (as shown in Table S1). To solve this issue, the nozzle was first moved up, shifted in parallel to the build plate by holding the paste extrusion, and then pulled down to print. These steps are depicted in Figure S3a and added to the G-code shown in Figure S3b.

Since the carbon or Ag/AgCl paste is a non-Newtonian fluid, its viscosity causes a retarded response to the applied force. This property resulted in the paste continuing to flow from the tip during the transition from one printing point to another, even though the syringe pump had stopped pushing. The accumulation of paste at the tip led to inaccuracies in the printing dimensions due to the excessive paste output (as shown in Table S1). Inaccurate printing dimensions, such as the uneven thickness of a layer, could affect the printing of a second layer on top of it. Retraction at the syringe pump did not resolve the issue due to the paste’s delayed response to force. Therefore, as illustrated in Figure S4a, the printing head was moved to a sponge placed at the edge of the build plate to remove the accumulated paste at the needle tip before moving to the new printing position. The modified G-code and a photo of the 3D printer with axis labels and sponge position are presented in Figure S4b and Figure 1a, respectively.

### 3.2. Thicknesses of the 3D-Printed Electrodes

The thicknesses of the PMMA (substrate), printed Ag/AgCl (first layer), and carbon (second layer) were measured by a micrometer screw gauge, and the calculated mean thicknesses and standard deviations are shown in Table 1.

Table 1 shows that there were large variations in the thicknesses of the electrodes from chip to chip. Because the PMMA only shows a standard deviation of 6.3 μm, compared with the ideal gap of 0.1 mm between the nozzle tip and the substrate, the influence of the PMMA thickness variation on the calibrated distance was only 6.3%. One major factor contributing to the large variations in layer thicknesses was the imperfect manual leveling of the build plate, which, due to human eyesight and manual operation, could introduce an error of ±0.05 mm. The standard deviations for Ag/AgCl and carbon layers were almost the same, which implies that the thickness deviations could be introduced by the initial build plate leveling calibration, rather than during printing. If the deviations were due to the printing process, the second layer on top of the first layer would have larger deviations than the first layer. Introducing real-time automatic build plate leveling during the 3D printing process could significantly reduce thickness variations caused by manual calibration.

Another factor for thickness variation could be related to the high viscosity of the paste and a nozzle size of 0.25 mm. Compared with layer thicknesses of 0.055 mm and 0.035 mm for Ag/AgCl and carbon, the nozzle size was quite large. The difficulty in using a standard 3D printer to print electrodes lies in the need for extra force to extrude high-viscosity paste precisely at the nozzle. While inkjet printing can achieve this, it only works for low-viscosity liquids, thus requiring a sufficiently large nozzle for thicker pastes. In such cases, it is challenging to balance the size accuracy of the printed pattern with the smooth extrusion of the paste. Diluting high-viscosity paste with an appropriate solvent is a possible solution; however, this approach is complex, and the post-treatment process for the printed EC chips would need to be optimized.

A suitable electrode thickness for an EC chip should range from ten to hundreds of micrometers. For example, commercially available Zensor and PalmSens screen-printed electrodes have thicknesses of 10 μm and 350–380 μm, respectively [51,52]. Research indicates that electrodes with lower thicknesses exhibit low current values and high resistance, while higher current values are recorded for electrode thicknesses starting from 60 μm onwards [53]. Thus, the thicknesses of our printed electrodes were within a satisfactory range for sensing, although they were not very thick. It is possible to change the thicknesses of the printed electrodes by printing at different speeds or printing the Ag/AgCl and carbon pastes with multiple layers. However, for multi-layer printing, each layer must be cured first (by heating the substrate or leaving it to air dry) before the second layer is printed.

### 3.3. Insulating Layer

The insulating layer prevents the conducting path from participating in the EC reactions during the test. Two insulating materials were deployed and tested: the double-sided adhesive tape and the gray dielectric paste. Both materials were formed by polymer materials that had high electrical resistance, and we expected them to have similar functions for insulation.

**Figure 3:**
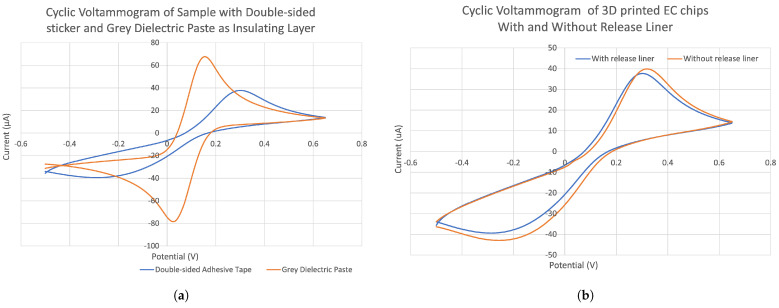
Recorded CVs of (**a**) sample chips with double-sided adhesive tape and gray dielectric paste as the insulating layer, (**b**) 3D-printed electrodes with and without release liner. For the CV test, 100 μL of 5 mM K_3_Fe(CN)_6_ in 0.1 M KCl solution was pipetted on an EC chip, scanned at a rate of 0.05 V/s.

Figure 3a shows that the peak separation of the CV curves for the gray dielectric paste chip was smaller than that for the double-sided adhesive tape chip. In addition, the heights for both the reduction and oxidation peaks of the gray dielectric paste chip were higher than those of the double-sided adhesive tape chip. The results confirm that the gray dielectric paste-insulated chip has better reversibility and sensitivity than the double-sided adhesive tape-insulated chip.

Figure 3b further indicates that the performance of the double-sided adhesive tape was poor, regardless of whether the top polyester release liner was removed. We are not very clear about the mechanisms causing this effect. However, we hypothesize that the reason might be that the gray dielectric D2070423P5 forms a cover coat that minimizes the pin-holing effect [48]. The double-sided tape might be porous, which could fail to provide sufficient insulation among the electrodes and also inadequately define the working electrode area.

### 3.4. Cyclic Voltammetry of Fabricated EC Chip

**Figure 4:**
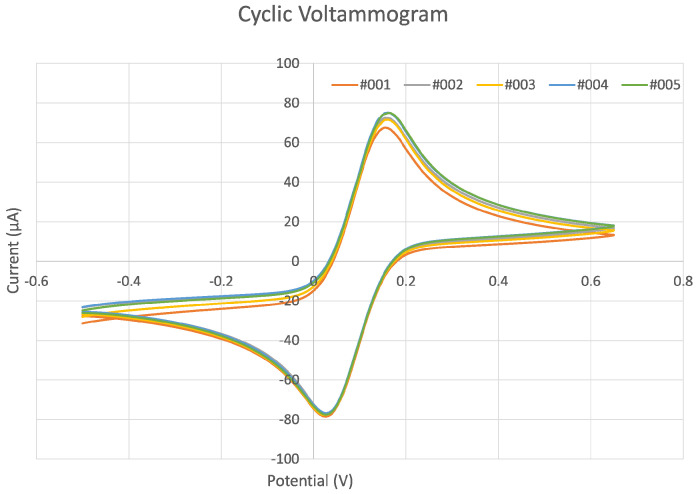
CV curve for the reduction of 5 mM K_3_Fe(CN)_6_ in 0.1 M KCl on a 3D-printed EC chip with 5 consecutive measurements, scanned at a rate of 0.05 V/s.

CV curves obtained from the redox reactions of Fe(CN${)}_{6}^{3-}$ and Fe(CN${)}_{6}^{4-}$ in KCl at the working electrode of a fabricated EC chip are shown in Figure 4. When the potential was scanned from −0.50 V to +0.65 V, the Fe(CN${)}_{6}^{3-}$ was reduced to Fe(CN${)}_{6}^{4-}$ and the cathodic peak occurred at 0.161 V. When the scan was reversed, the Fe(CN${)}_{6}^{4-}$ oxidized back to Fe(CN${)}_{6}^{3-}$ and the anodic peak occurred at 0.025 V. A total of five scans were repeated on the same EC chip. The presence of only one peak on each side indicates that only one species was involved in the reduction and oxidation reactions at the working electrode. The peak positions of the five CV cycles remain constant. The ratio of the anodic to the cathodic current peak was 0.95 (which is quite close to 1), and the separation between the peaks was 0.136 V for 5 mM K_3_Fe(CN)_6_ in 0.1 M KCl solution. Although the ideal peak separation for a one-electron CV process is theoretically 0.059 V [3], our results are very close to those of the commercial carbon and silver/silver chloride screen-printed chips from DropSens, which showed a peak separation of 0.126 V tested with 10 mM K_3_Fe(CN)_6_ in 0.1 M KCl solution (refer to the Figure S5, Supplementary Materials).

The 3D-printed EC chips or screen-printed EC chips are ohmically uncompensated EC cells; thus, the CV detection is not perfect. The theoretical simulation revealed that screen-printed EC chips (which have the same structure as our 3D-printed cells) had a 0.12 V peak separation for CV curves of 5 mM Fe(CN${)}_{6}^{3-}$/Fe(CN${)}_{6}^{4-}$, while this number was reduced to 0.11 V for 10 mM Fe(CN${)}_{6}^{3-}$/Fe(CN${)}_{6}^{4-}$ [54]. Therefore, our 3D-printed EC chip has good reversibility and similar performance in CV tests to the screen-printed EC chip from DropSens. The literature indicates that this peak separation is primarily determined by the carbon paste material, the formulation of which is a commercial secret [55,56].

### 3.5. Differential Pulse Anodic Stripping Voltammetry with Standard Solution

**Figure 5:**
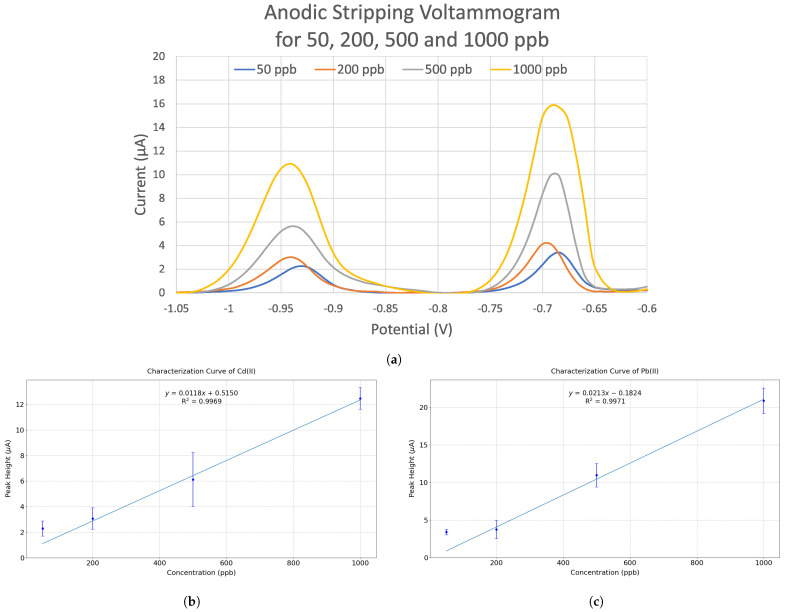
(**a**) Voltammogram graphs of DPASV tests with standard solutions that contained 50 ppb, 200 ppb, 500 ppb, and 1000 ppb of both Pb(II) and Cd(II) in the 0.1 M acetate buffer solution at pH = 4.5, and the calibration curves of (**b**) Cd(II) and (**c**) Pb(II).

DPASV tests were carried out for three 3D-printed EC chips, with a standard solution containing equal concentrations of Pb(II) and Cd(II) in the range of 50 ppb to 1000 ppb in 0.1 M acetate buffer at pH = 4.5. A representative of DPASV voltammogram is shown in Figure 5a; the peaks at around −0.70 V and −0.95 V specify the existence of Pb(II) and Cd(II), respectively. The heights of the peaks increased with the concentrations of Pb(II) and Cd(II), correlating to the increments of Pb and Cd deposited on and then stripped from the working electrode.

We processed the data by first deducting the background of each measurement, and then identifying the peak heights for Pb(II) or Cd(II) (see Figure S6, Supplementary Materials). The standard deviation for the peak heights of the three chips at the same Pb(II) or Cd(II) concentration was calculated as the error bar (whose positive or negative error equals the standard deviation). The calibration curves plotted in Figure 5b,c have a linear range for Pb(II) and Cd(II) detection from 200 to 1000 ppb at an R-square value of 0.997. The characterization curves plotted from 200 to 1000 ppb make sense because both curves imply that the current peak signal will be close to zero when the heavy metal ion concentration is zero. However, the current peak for 50 ppb of Pb(II) or Cd(II) in Figure 5a is significantly more anodic than other concentrations, and the 50 ppb point does not belong to the linear range of the characterization curve in Figure 5b or Figure 5c. Therefore, we are only confident that the EC chip is able to reliably measure Pb(II) and Cd(II) at a concentration of 200 ppb. Due to the lack of measurement data for Pb(II) and Cd(II) at even lower concentrations and concentrations between 50 and 200 ppb, we are not able to identify the detection limit of this EC chip at this moment. In the future, more experiments will be conducted to obtain the full sensing range (from the detection limit to the highest detectable concentration before signal saturation) of the 3D-printed EC chips for Pb(II) and Cd(II) concentrations. We tested and found that the commercial EC chips screen-printed with carbon and silver/silver chloride from DropSens can detect 25 ppb of both Pb(II) and Cd(II); the linearities were similar to the 3D-printed EC chips, but the error bars were smaller. Overall, the 3D-printed EC chips have a lower sensitivity to Pb(II) and Cd(II) detections.

The relatively large error bars in Figure 5b,c reveal the degree of inconsistency of the peak height in each test. This inconsistency could be attributed to the thickness variations in the printed electrodes as well as some deviations in surface roughness from chip to chip. The thickness and surface roughness inconsistencies of the working electrode relate to the printing mechanism and the build plate leveling. As the electrodes are printed in a raster pattern using a 3D printer, imperfect build plate leveling could cause the raster pattern to be printed with varying roughness. Different working electrode thicknesses could result in varying electrode resistances, and their surface roughness could affect the electroactivity of the analytes on the working electrode [11,53]. As a result, the signal received could vary from chip to chip. However, the rough surface of the electrodes is beneficial for enhancing the sensitivity of EC detection because the surface for EC reaction is increased.

The sensitivity of the EC detectors is influenced by the background current, which comprises the charging current and the faradic current from the redox reactions of impurities or reactions of the electrode itself. The DPASV sampling method that we used largely reduced the amount of the charging current compared to linear sweep voltammetry; thus, the relatively high faradic current could be the main background current. The impurities are inevitable, which could be due to the small amount of dissolved oxygen and the salt used as a supporting electrolyte. Other than that, when a bare electrode is used, surface reactions on the electrode, such as redox reactions of surface functionalities at the carbon electrode surface, may contribute to the faradic background current too [1]. This relates to the surface stability of the working electrode, and a higher annealing temperature of the EC chip could improve the detection limit of the chip. The EC chip can be printed on a ceramic substrate instead of the PMMA substrate so that the chip can be annealed at a much higher temperature to increase the working and reference electrodes’ stability and adhesion on the substrate.

## 4. Conclusions

EC detection is a good candidate for on-site tests due to its minimal sample preparation, low cost, and rapid testing. In this work, we demonstrated a method of fabricating EC chips by 3D printing two layers of paste, which offers greater flexibility and lower costs than the prevailing commercial screen printing technology used for EC chip fabrication. The fabrication process was relatively simple for leveling the printing build plate, filling the paste, printing the silver/silver chloride paste on a PMMA substrate, overlaying it with carbon paste, annealing the chip at 70 °C for 1 h, and applying the dielectric paste for insulation. With engineering issues such as needle clogging, whiskers in the patterns, and needle cleaning resolved, high-quality EC chips were produced on PMMA substrates. Compared with screen printing, the 3D printing of electrodes is a cleaner and less tedious process.

The CV test with a K_3_Fe(CN)_6_/KCl solution shows that each 3D-printed EC chip has relatively high reversibility and repeatability for multiple tests. By the DPASV test, the 3D-printed EC chip can quantitatively detect Pb(II) and Cd(II) standard solutions in concentrations ranging from 200 to 1000 ppb. The test performance of the 3D-printed EC chips was comparable to that of commercially available screen-printed EC chips.

The imperfect leveling of the build plate was found to be the main factor causing the chip-to-chip variations of the EC peak response. A laser positioning sensor can be integrated into the 3D printer for auto-alignment of the build plate, keeping an accurate 0.1 mm gap between the needle tip and the substrate to achieve higher consistency in the 3D-printed EC chips. It is also anticipated that using a ceramic substrate, which is commonly adopted in commercial screen-printed EC chips, would allow a higher annealing temperature to be applied, further stabilizing the 3D-printed electrodes and achieving a lower detection limit for the EC chips. With the development of multi-nozzle 3D printing technology, it is possible to mass-produce EC chips using pastes. The technologies demonstrated in this work, such as avoiding needle clogging and removing whiskers and excessive paste, are viable and compatible with these mass fabrication tools. Furthermore, electrodes 3D-printed with pastes can be easily integrated with fluidic channels as disposable lab-on-a-chip devices.

## Figures and Tables

**Table 1:** Mean values and standard deviations of the thicknesses of the substrate, the first layer, and the second layer of the printed electrode.

Material	PMMA (Substrate)	Ag/AgCl (First Layer)	Carbon (Second Layer)
Mean Values (mm)	1.00	0.055	0.035
Standard Deviation (mm)	0.0063	0.023	0.023

## Data Availability

The datasets generated during and/or analyzed in this study are available from the corresponding author upon reasonable request.

## Supplementary Materials

The following supporting information can be downloaded at https://www.mdpi.com/article/10.3390/s24092844/s1, Figure S1: The filling of carbon paste into the syringe barrel. Figure S2: Photo of the fabricated electrodes with (a) double-sided sticker and (b) grey dielectric paste as the insulating layer. Figure S3: (a) Schematic diagram of additional movement added into the G-code for removing the whiskers. (b) G-code before and after the addition of extra movements. Figure S4: The cleaning step added to remove the excessive paste accumulated on the print head. Figure S5: Recorded cyclic voltammogram of commercial carbon chip using 10 mM K_3_Fe(CN)_6_ in 1.0 M KCl solution. Figure S6: Voltammograms of DPASV tests with baseline deducted for Pb(II) and Cd(II) in 0.1 M acetate buffer solution with pH = 4.5. Table S1: Engineering problems encountered and solved during the 3D printing of EC chips.

## Abbreviations

The following abbreviations are used in this manuscript:

DPASV	differential pulse anodic stripping voltammetry
CV	cyclic voltammetry
EC	electrochemical
PMMA	poly(methyl methacrylate)
PTFE	polytetrafluoroethylene
