# Supplementary material for: Adaptive Fabrication of Electrochemical Chips with a Paste-Dispensing 3D Printer
Source: Sensors (Basel). 2024 Apr 29;24(9):2844. doi: 10.3390/s24092844 (PMC11086071; doi:10.3390/s24092844)
Supplement: Supplementary file 1 [file sensors-24-02844-s001.zip › sensors-2943046-supplementary update.pdf]

Supplementary Materials

Adaptive Fabrication of Electrochemical Chips with a Paste-Dispensing 3D Printer

Ten It Wong [1,†], Candy Ng [2,†,‡], Shengxuan Lin [3,‡], Zhong Chen [2] and Xiaodong Zhou [1,*]

[1] Institute of Materials Research and Engineering, A*STAR (Agency for Science, Technology and Research), 2 Fusionopolis Way, #08-03, Innovis, Singapore 138634, Singapore; wongti@imre.a-star.edu.sg
[2] School of Materials Science & Engineering, Nanyang Technological University, Block N4.1, Nanyang Avenue, Singapore 639798, Singapore; candyng107@gmail.com (C.N.); aszchen@ntu.edu.sg (Z.C.)
[3] Residues and Resource Reclamation Centre (R3C), Nanyang Environment and Water Research Institute, Nanyang Technological University, 1 Cleantech Loop, Clean Tech One, Singapore 637141, Singapore; lin.shengxuan@gmail.com
* Correspondence: donna-zhou@imre.a-star.edu.sg
† These authors contributed equally to this work.
‡ The authors were with the indicated affiliation during the conduction of experiments in this work.

List of Figures

S1 Filling of carbon paste into the syringe barrel. s2
S2 Photo of the fabricated electrodes with (a) double-sided stickers and (b) grey dielectric paste as the insulating layer.. . . . . s2
S3 (a) Schematic diagram of additional movement added into the G-code for removing the whiskers. (b) G-code before and after the addition of extra movements. s4
S4 The cleaning step added to remove the excessive paste accumulated on the print head. s5
S5 Recorded cyclic voltammogram of commercial carbon chip using 10 mM $K_3Fe(CN)_6$ in 1.0 M KCl solution. s5
S6 Voltammograms of ASV tests with baseline deducted for Pb(II) and Cd(II) in 0.1 M acetate buffer solution with pH = 4.5. . . . . s6

List of Tables

S1 Engineering problems encountered and solved during the 3D printing of EC chips. s3

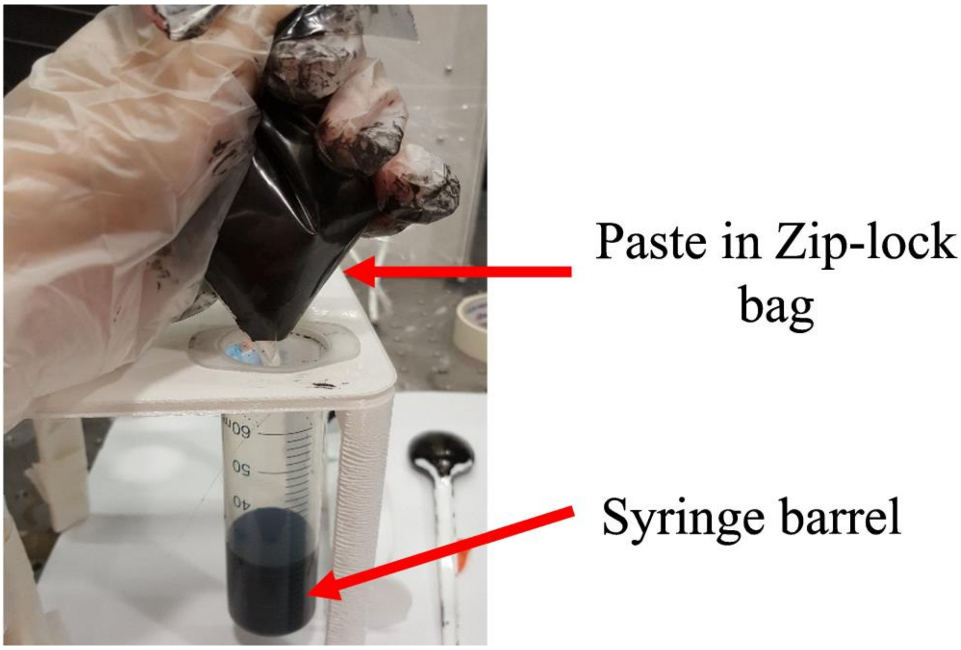

Figure S1. The filling of carbon paste into the syringe barrel. To avoid bubbles, the paste is squeezed intothe syringe barrel through a 2 mm diameter small hole cut on the corner of a zip-lock bag.

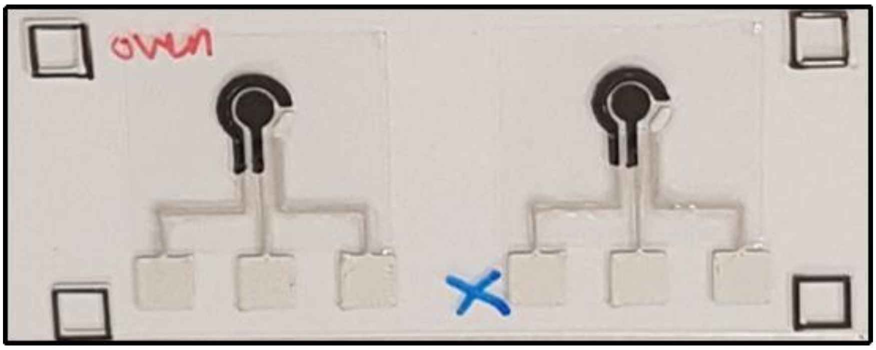

(a)

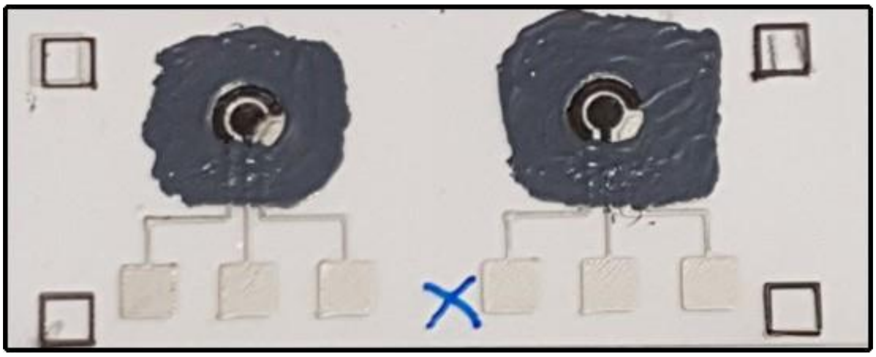

(b)

Figure S2. Photo of the fabricated electrodes with a) double-sided stickers and b) grey dielectric paste as theinsulating layer.

Table S1. Engineering problems encountered and solved during the 3D printing of EC chips.

Issues	Solutions	Examples
Poor levelling caused scratching on the substrate or disconnected lines	Carefully aligned, and thegap between the nozzle and the PMMA substratewas set to 0.1 mm	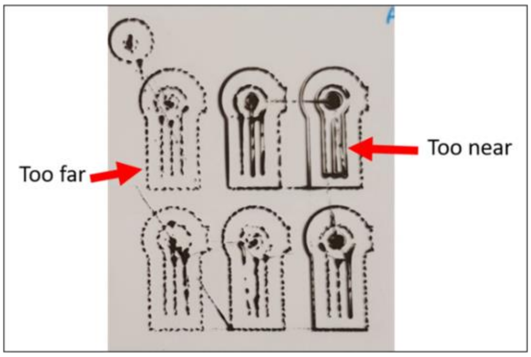
Bulge of the tube with pressure accumulation	1. Replace the polyethylene tube with a PTFE tube<br>2. Clean thoroughly and seal tightly forreused needles	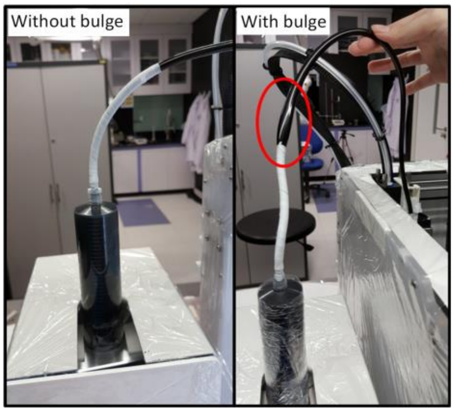
Whiskers on the printed chip	The nozzle was first moved up, held the paste extrusion, and then pulleddown to print (Figure S3)	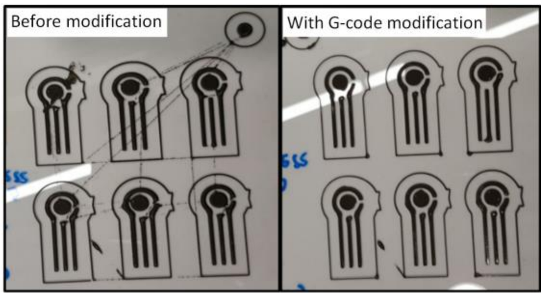
Inaccurate printing dimension due to an excessive amount of paste	Moved to a sponge to remove the accumulated paste at the needle tip (Figure S4)	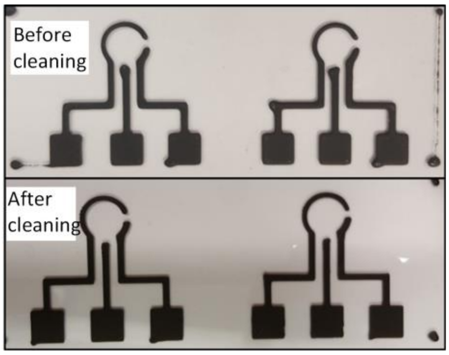

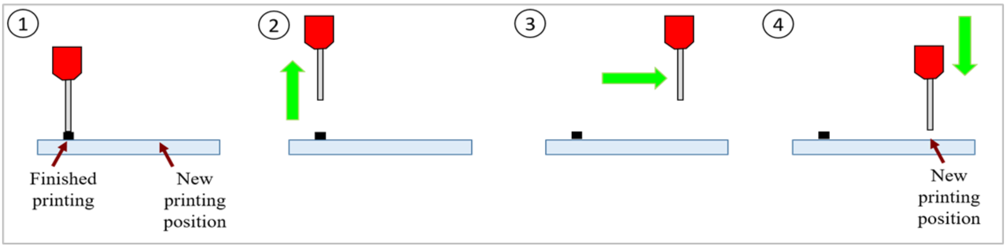

(a)

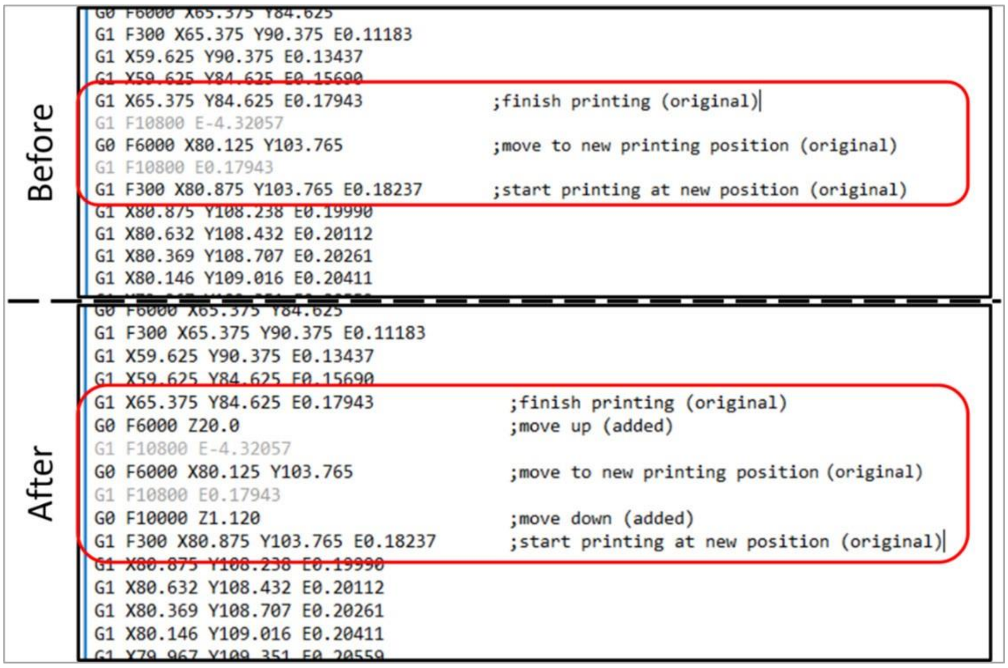

(b)

Figure S3. (**a**) Schematic diagram of additional movement added into the G-code for removing the whiskers. (**b**) G-code before and after the addition of extra movements.

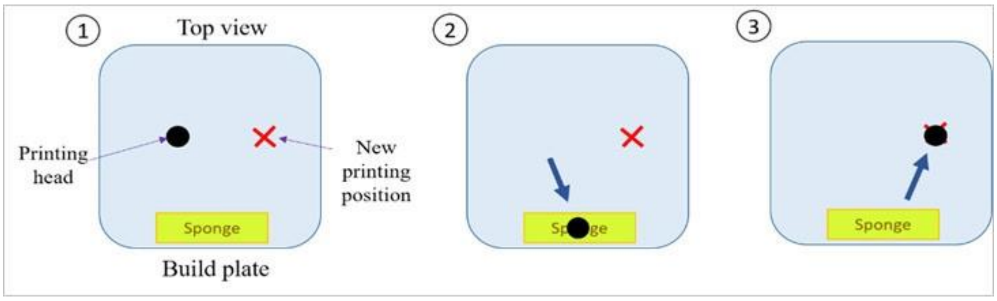

(a)

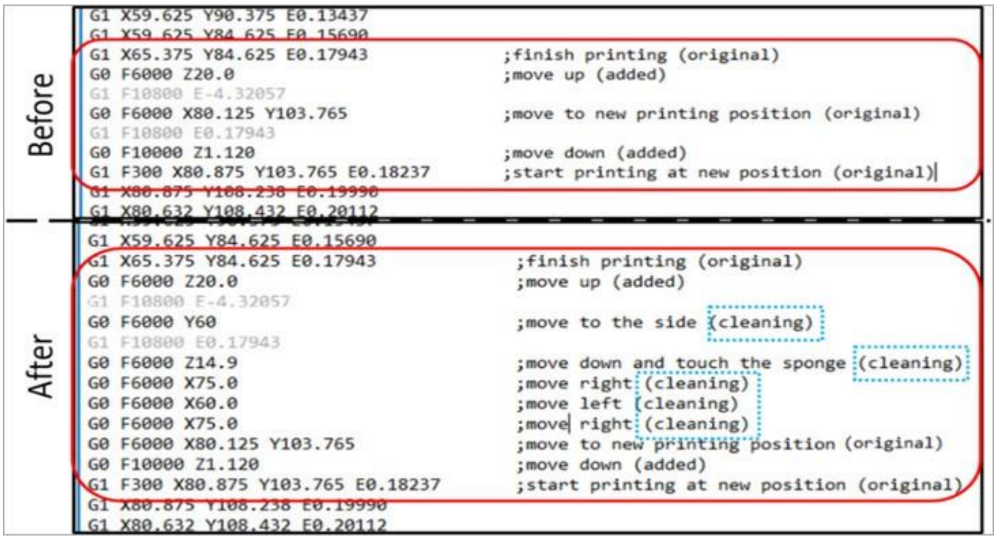

(b)

Figure S4. The cleaning step added to remove the excessive paste accumulated on the print head: (**a**) thecleaning step added and (**b**) the modification of the G-code.

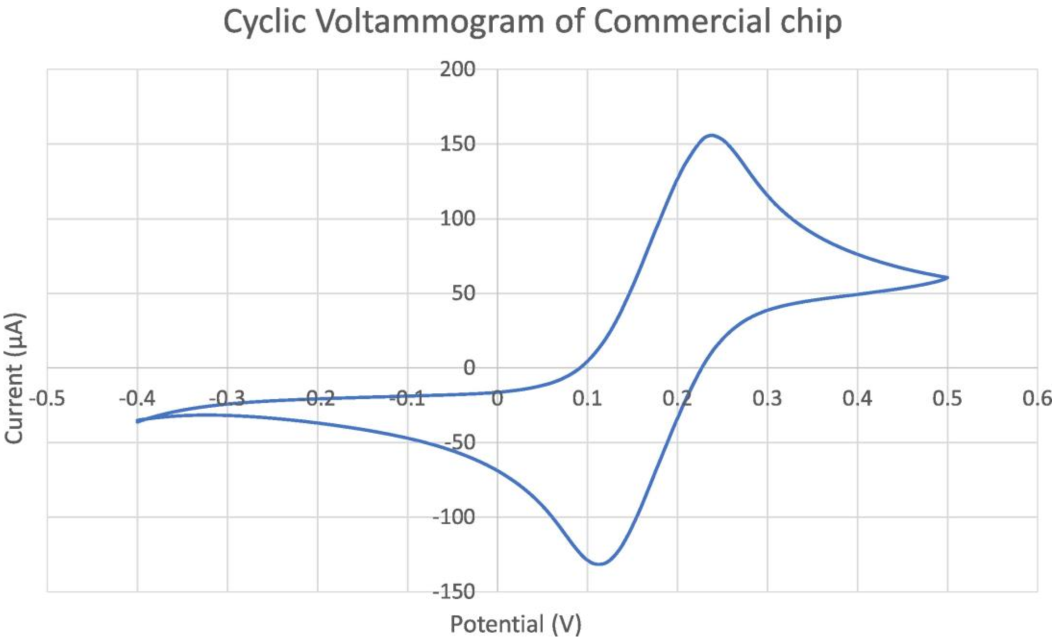

Figure S5. Recorded cyclic voltammogram of a commercial carbon chip using 10 mM $K_3Fe(CN)_6$ in 1.0 MKCl solution.

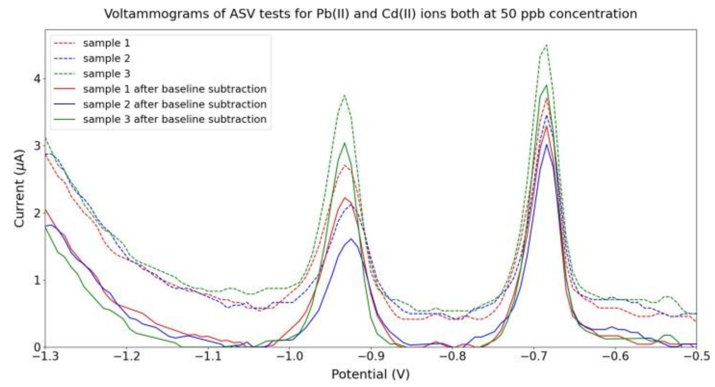

(a)

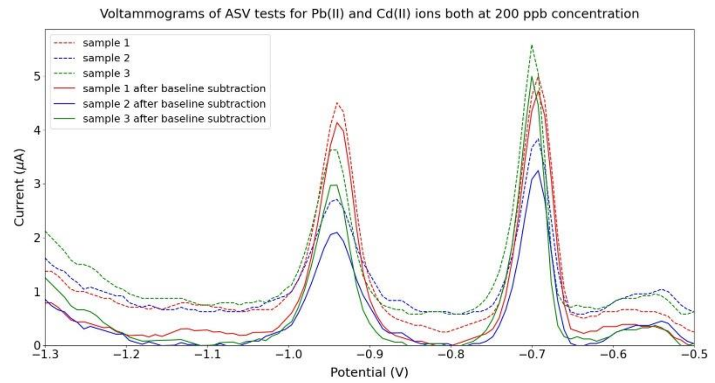

(b)

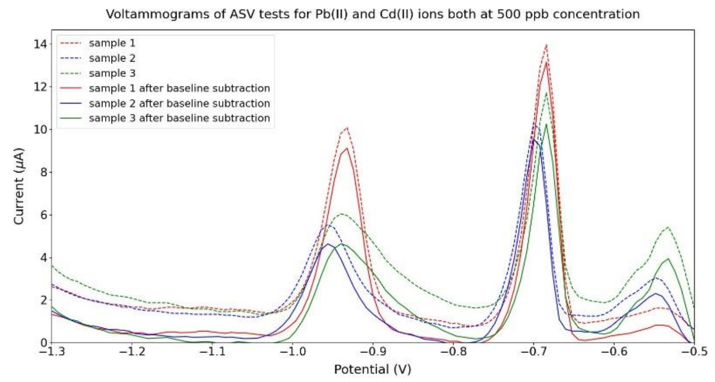

(c)

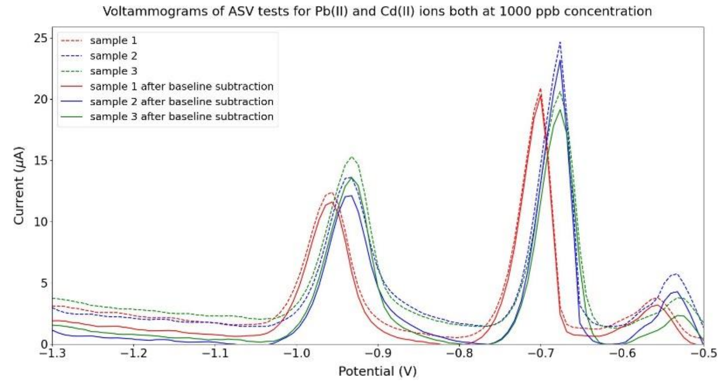

(d)

Figure S6. Voltammograms of ASV tests with the baseline deducted for Pb(II) and Cd(II) in 0.1 M acetate buffer solution with pH = 4.5, at the concentrations of (a) 50 ppb, (b) 200 ppb, (c) 500 ppb, and (d) 1000 ppb for both ions.
